# Psychometric Properties of Parent–Child (0–5 years) Interaction Outcome Measures as Used in Randomized Controlled Trials of Parent Programs: A Systematic Review

**DOI:** 10.1007/s10567-019-00275-3

**Published:** 2019-02-07

**Authors:** Nicole Gridley, Sarah Blower, Abby Dunn, Tracey Bywater, Karen Whittaker, Maria Bryant

**Affiliations:** 1 Department of Health Sciences, University of York, York, UK; 2 School of Nursing, University of Central Lancashire, Preston, UK; 3 Clinical Trials Research Unit, Leeds Institute of Clinical Trials Research, University of Leeds, Leeds, UK; 4 Present Address: School of Education, Leeds Beckett University, Leeds, LS6 3QQ UK

**Keywords:** Observation, Parent–child relationships, Systematic review, COSMIN, Psychometric properties

## Abstract

This systematic review sought to identify observational measures of parent–child interactions commonly implemented in parenting program research, and to assess the level of psychometric evidence available for their use with this age group. Two separate searches of the same databases were conducted; firstly, to identify eligible instruments, and secondly to identify studies reporting on the psychometric properties of the identified measures. Five commercial platforms hosting 19 electronic databases were searched from their inception to conducted search dates. Fourteen measures were identified from Search 1; a systematic search of randomized controlled trial evaluations of parenting programs. For Search 2, inclusion/exclusion criteria were applied to 1327 retrieved papers that described the development and/or validation of the 14 measures identified in Search 1. Seventeen articles met the inclusion criteria, resulting in five observational measures for the final review. Data were extracted and synthesized using the COSMIN rating system to describe the methodological quality of each article alongside the overall quality rating of the psychometric property reported for each measure using the Terwee checklist. Measure reliability was categorized into four domains (internal consistency, test-re-test, inter-rater, and intra-rater). Measure validity was categorized into four domains (content, structural, convergent/divergent, and discriminant). Results indicated that the majority of psychometric evidence related to children aged from birth the three with internal consistency, inter-rater reliability, and structural validity the most commonly reported properties, although this evidence was often weak. The findings suggest further validation of the included measures is required to establish acceptability for the whole target age group.

**Electronic supplementary material:**

The online version of this article (10.1007/s10567-019-00275-3) contains supplementary material, which is available to authorized users.

Behavioral difficulties and social and emotional problems are the most common reasons for clinical assessment amongst 2–5-year olds (Keenan and Wakschlag 2000). Difficulties in these domains are relatively stable over time, with approximately 50% of all 2–3-year olds with problematic behavior receiving a diagnosis of a behavioral disorder 42–48 months later (Alink et al. 2006). Diagnosed children are at a greater risk for more severe problems by the time they reach school age (Shaw et al. 2003), with persistent behavior problems contributing to impairments in social and cognitive development (Stams et al. 2002; Stright et al. 2008), increased inter-personal conflicts with peers (Menting et al. 2011), and low levels of academic competence and performance (Stright et al. 2008). In the longer term, these children are more likely to use mental health services (Essex et al. 2009) with estimates suggesting that an additional £70,000 per individual is needed to fund services by the time they reach 30 years old (Scott et al. 2001). It is widely accepted that the development of psychopathology is best understood in the context of early parent–child interactions and that precursors can be detected during infancy (Skovgaard et al. 2007, 2008). Consequently, early assessment and identification is paramount to ensuring the best outcomes for all children and families. Some observational measures can be used to identify children and families in need of intervention, to monitor their progress, and to evaluate programs as part of research. However, they must satisfy stringent psychometric criteria of reliability and validity to ensure assessment accuracy in order that families receive relevant offers of support and reliable monitoring of their progress.

The quality of early parent–child (birth to 5 years) interactions provide the foundation for all future social interactions and are considered an important component for conceptualizing and assessing behavioral and emotional difficulties in infancy (Zeanah 2009). For example, research indicates that sensitive and responsive parenting that is tailored to an infant’s developmental needs predicts secure attachment (Kim et al. 2017), social and emotional competence (Leerkes et al. 2009; Raby et al. 2015), advanced cognitive abilities (Bernier et al. 2010, 2012; Evans and Porter 2009), and good quality language outcomes (Costanstini et al. 2011; Gridley et al. 2016; Hudson et al. 2015). In contrast, children exposed to less sensitive or responsive parenting, or to repetitive and punitive caregiving, are at greater risk for developmental disadvantage by 16 years (Bender et al. 2007) unless effective treatments and interventions are received (Barlow et al. 2016).

Parent programs are the preferred preventative intervention/treatment for childhood behavior, social, and emotional problems (Bywater 2017). There is an increasing awareness amongst researchers and practitioners that the process of identifying, assessing, and evaluating should be supported by the use and implementation of robust measures that provide reliable and valid outcomes (Arora et al. 2016). Unfortunately, many measures used routinely with older children are adopted for use with younger age groups without consideration as to whether they are acceptable or psychometrically sound (Pontoppidan et al. 2017). As a result, commonly used measures in research and practice may be unfit for purpose and there is a need to re-assess the level of psychometric evidence when used with this younger age group.

Observational methods are considered the gold standard assessment of parent–child interaction (Hawes and Dadds 2006) because they provide objective, fine-grained, details of the relationship that may occur without awareness (Wysocki 2015). In contrast to other assessment measures (i.e., questionnaires) observational assessments can identify both the strengths and difficulties that occur during early dyadic interactions that might influence the trajectory of a child’s development (Bennetts et al. 2016), and they directly measure behavior as it happens in real time (Dishion et al. 2017). Moreover, as most observations can be conducted in the home without being prescriptive (Bagner et al. 2015) they are often regarded as essential to a multi-component assessment which provides a comprehensive evaluation of the caregiving environment (Bagner et al. 2012; Aspland and Gardner 2003). As supporting parent–child interaction is often the key goal of early intervention programs (Gottwald and Thurman 1994) the use of observational tools as outcome measures is now seen by many as being integral to understanding change at a meaningful level (NICE 2017).

There are a number of observational measures available to researchers and practitioners to assess early parent–child interactions, but these measures target a broad range of constructs (i.e., dyadic synchrony, maternal responsivity/sensitivity, emotional availability, affect, learning support, intrusiveness), and subsequently utilize different units for coding target behavior (Aspland and Gardner 2003; Lotzin et al. 2015). Coding schemes are typically classified into two categories; macro or micro (Dishion et al. 2017; Rosenberg et al. 1986). Macro observations utilize broad categories (i.e., responsivity/sensitivity) to summarize substantial amounts of information into usable components. These schemes typically utilize global ratings to make judgements based on the number of acts observed over a period of time, and as a consequence such schemes require less rigorous training in order for users to become reliable (Rosenberg et al. 1986). In contrast, micro observational schemes encompass specific and narrowly defined categories, which capture moment-to-moment behaviors as miniature chunks of information either via interval coding, or continuous recording (Dishion et al. 2017; Morawska et al. 2014; Rosenberg et al. 1986). Due to their complexity micro observational schemes require extensive training, but it is argued that these measurements of parent–child dynamics are more sensitive to change following intervention (Dishion et al. 2017; Morawska et al. 2014). Due in part to methodological variation between measures, there is little agreement in the literature as to which is accepted as the single standard for measuring parent-infant interaction (Lotzin et al. 2015). Consequently, when researchers and practitioners are selecting the most appropriate measure to be used for their purpose it is argued that careful consideration of a measure’s reliability and validity should be taken into account (Lotzin et al. 2015; Rosenberg et al. 1986).

According to the COnsensus-based Standards for the selection of health Measurement Instruments (COSMIN; de Vet et al. 2015; Terwee et al. 2007) reliability is defined as the degree to which a measure is free from measurement error. The extended definition distinguishes between four reliability assessments that can be determined for most observational measures. *Internal consistency* refers to the degree of interrelatedness among items of a given observational tool, and only lends itself to observational tools that utilize non-dichotomous recording methods (i.e., frequency counts or Likert scales). *Test–re-test reliability* seeks to establish a measure’s stability over time and can be performed on all observational tools where data are available at two timepoints. Finally, *inter-* and *intra-rater reliability* are two assessments of coder/rater consistency. Inter-rater assesses scores from different people at the same time, whilst intra-rater assesses scores from the same person at different times. Both inter- and intra-rater reliability are easily applied across all observational coding schemes irrespective of recording method or number of observations and are the most commonly used psychometric assessment for observational measures (Aspland and Gardner 2003).

The COSMIN states validity is the degree to which a measure truly measures the construct it purports to measure. The extended definition distinguishes three types of validity that can be determined for most observational tools. *Content* validity is the degree to which a measure is an adequate reflection of the construct that it intends to measure. This level of validity is typically determined by agreement amongst experts in the field during coding scheme construction. *Criterion* validity is the degree to which scores of a measure are an adequate reflection of the gold standard. Given that there is not one single standard for measuring parent–child interaction this aspect of validity is particularly difficult to determine for most observational tools. Finally, *construct validity* is the degree to which the scores of a measure are consistent with the hypotheses. Construct validity is typically viewed as an umbrella term to describe three aspects of a measures property that are particularly important for observational measures; *structural validity, hypothesis testing*, and *cross-cultural validity*. In terms of observational measures structural validity is the degree to which scores of a measure are an adequate reflection of the dimensionality of the construct to be measured typically assessed using factor analysis to confirm composite variables. Hypothesis testing is the degree to which relationships between scores on one measure are sufficiently related (convergent) or unrelated (divergent) to scores on other instruments measuring similar or dissimilar constructs, or different groups of patients (discriminative). Finally, cross-cultural validity is the degree to which performance of the items on a translated or culturally adapted instrument reflect the performance of items in the original version. In addition to reliability and validity, the COSMIN describes a further dimension of a measure’s psychometric properties; responsiveness. Responsiveness is defined as the ability to detect change following intervention and is critical to a measures ability to be used as an outcome measure in research and practice.

Previous reviews have indicated that the most commonly reported psychometric properties for observational measures of parent–child interactions tend to be aspects of reliability, whereas validity is under-reported (Aspland and Gardner 2003). Furthermore, not all components of reliability or validity are tested. For example, a non-systematic review (Munson and Odom 1996) indicated that whilst 94% of the 17 rating scales developed to measure parent-infant interaction from birth to 3 years reported on at least one form of reliability, only 29% provided both internal consistency and inter-rater agreement estimates. In terms of validity, 94% of measures reported evidence for at least one type of validity. Conversely, Bagner et al. (2012) indicated that of the four observational measures reviewed for the detection of emotional and behavioral problems in infancy (birth to 2 years) all reported on and evidenced at least one aspect of reliability and one aspect of validity. Whilst internal consistency and inter-rater reliability were the more commonly reported constructs of reliability, convergent, and discriminative or divergent validity were the most commonly reported aspects of validity. Locke and Prinz (2002) identified 33 observational tools for use with parents and their children aged from 1 to 18 years, with all but one reporting on at least one aspect of reliability and all but three reporting on one aspect of validity. Despite the encouraging findings, there is little information relating to the specific dimensions of reliability assessed, or indeed what the comparators for validation were.

More recent systematic reviews (Hurley et al. 2014; Lotzin et al. 2015; Perrelli et al. 2014) also found that results regarding measurement reliability (for use with children up to 18 years) are generally well reported, yet evidence for validity is scarce. For example, Lotzin et al. (2015) indicated that only 37.5% of the 24 reviewed measures for children under 12 months had supporting evidence of content validity and 66.6% of measures reported evidence for structural validity. Moreover, whilst 15 measures did evidence convergent validity overall the authors failed to find evidence across all five domains of validity, with less than 50% providing evidence across just four domains. For observational tools that focus specifically on nurturing behaviors (for parents of children aged 1–18 years) Hurley et al. (2014) identified that only one of three measures reported content validity, whilst the other two reported on only two dimensions of reliability with relatively acceptable levels.

Despite limitations of earlier reviews (e.g., search strategies and data synthesis methods), the findings highlight significant gaps in the knowledge of all psychometric properties for observational measures used to assess dyadic interactions across the age range of birth up to and including 5 years. Furthermore, it has been argued that there is a need to adopt a standardized method to synthesize findings from multiple reviews of measurement properties using predefined guidelines to allow for easy comparison across reviews (Lotzin et al. 2015; Terwee et al. 2016). As a result, a further systematic review to assess observational measures for parents and their children (aged 0–5 years) adopting a standardized method of synthesis was deemed worthwhile.

The current review had two aims. Firstly, we wanted to identify the most commonly reported observational outcome measures of parent–child interaction used in randomized controlled trial (RCT) evaluations of parenting programs delivered antenatally and/or for parents of children up to and including 5 years. Specifically, we were interested in observational measures that provided an assessment of parent–child interaction, including attachment, bonding, and/or maternal sensitivity. Secondly, we sought to identify and synthesize the current evidence base for each of the included measures psychometric properties via a second systematic search of the scientific literature.

The rationale for focusing specifically on commonly used measures within RCTs of parenting programs was twofold. Firstly, we wanted to find measures in robust evaluations because we assumed these would be the most reliable/valid tools. Secondly, we wanted to build on the consistency that already exists in the field since the parenting field has been well established for several decades. The purpose was to provide further evidence of the strengths and limitations of existing observational tools with the intention of being able to recommend particular tools for practice. Throughout the remainder of this review evidence for each of the included measures psychometric standing will be conceptually organized according to their reliability and validity using the terms and definitions applied by the COSMIN checklist (de Vet et al. 2015; Terwee et al. 2007).

## Method

This review had two distinct search stages. Search 1 identified RCTs of parenting programs for parents of children from the antenatal period up to the child’s sixth birthday published in the scientific literature. From these studies’ observational measures of parent–child interactions, which had been used to evaluate the intervention, were extracted. Measures which were identified as having been used in three of more of the retrieved RCTs were then included in Search 2. The purpose of Search 2 was then to identify papers describing the development and subsequent validation of these measures via an additional database search.

### Domain Map

In preparation for the systematic review two authors (TB and SB) in collaboration with an advisory group undertook a domain mapping exercise as recommended by Vaughn et al. (2013). The intention was to enable classification of identified outcome measures by population of interest. Outcome domains were mapped under three categories; parent, child, and dyadic. Search 1 only identified observational measures of dyadic outcomes. The results of which are reported within this review. The findings for the parent and the child domains are described in two companion reviews (Authors, in submission).

### Search 1: Identifying Tools Used in Parenting Program Research

#### Eligibility Criteria for Evaluation Studies

Search 1 was focused solely on identifying high-quality parent program evaluations i.e., RCT’s, consequently the literature search was restricted to peer-reviewed items. Included studies were: (1) primary research relating to the evaluation of a parenting program using an RCT design. Studies reported a randomly allocated treatment and comparison group (which was any comparator e.g., control, waiting list, other treatment). (2) Samples that included expectant parents, mothers and/or fathers or other types of primary carer, of children up to and including the age of 5 years (where the evaluation spanned a wider age range at least 80% of the participants had to meet this criteria). (3) Described a parenting program that was structured, manualized, delivered by a trained facilitator and consisted of three or more sessions that were designed to improve some aspect of child social and emotional wellbeing or behavior. (4) Reported on at least one relevant parent–child outcome (as determined by the domain mapping exercise) which had been developed and validated independently of the RCT. (5) A study published in the English language published within the period 1995–2015. Papers were excluded if they met the inclusion criteria but; (a) there was insufficient information to determine eligibility (where a scan of full text could not provide missing information), and (b) the manuscript was not available to download in full-text format from host University’s library, Endnote, Paperpile, or Google Scholar.

### Search Strategy for Obtaining Evaluation Studies

A total of five commercial platforms hosting 19 scientific databases were searched in November 2015 with only studies published after January 1995 included because of concerns about the design and reporting of studies before this date. Databases were all searched in English. An example of the search strategy used for retrieving relevant papers from each of the 19 databases is as follows;

parent* training* OR parent* program* OR parent* education OR parent* intervention* AND toddler OR infant OR pre*school OR bab*y OR child* OR pregnancy OR antenatal AND experimental OR randomi?ed controlled trial

The flowchart depicting article retrievals for Search 1 is shown in Online Resource Fig. 1. The databases searched to identify relevant articles were: Arts and Humanities Citation Index, ASSIA, British Nursing Index, CINAHL plus, Cochrane Library, Conference Proceedings Index, DARE, Econlit, EMBASE, ERIC, HTA, Maternity and Infant care Database [MIDIRS], MEDLINE Journal articles, NHS EED, Psycharticles, PsychInfo, Social Policy and Practice database [SOPP], Social Science Citation Index expanded, and Social Sciences Citation Index.

**Fig. 1:**
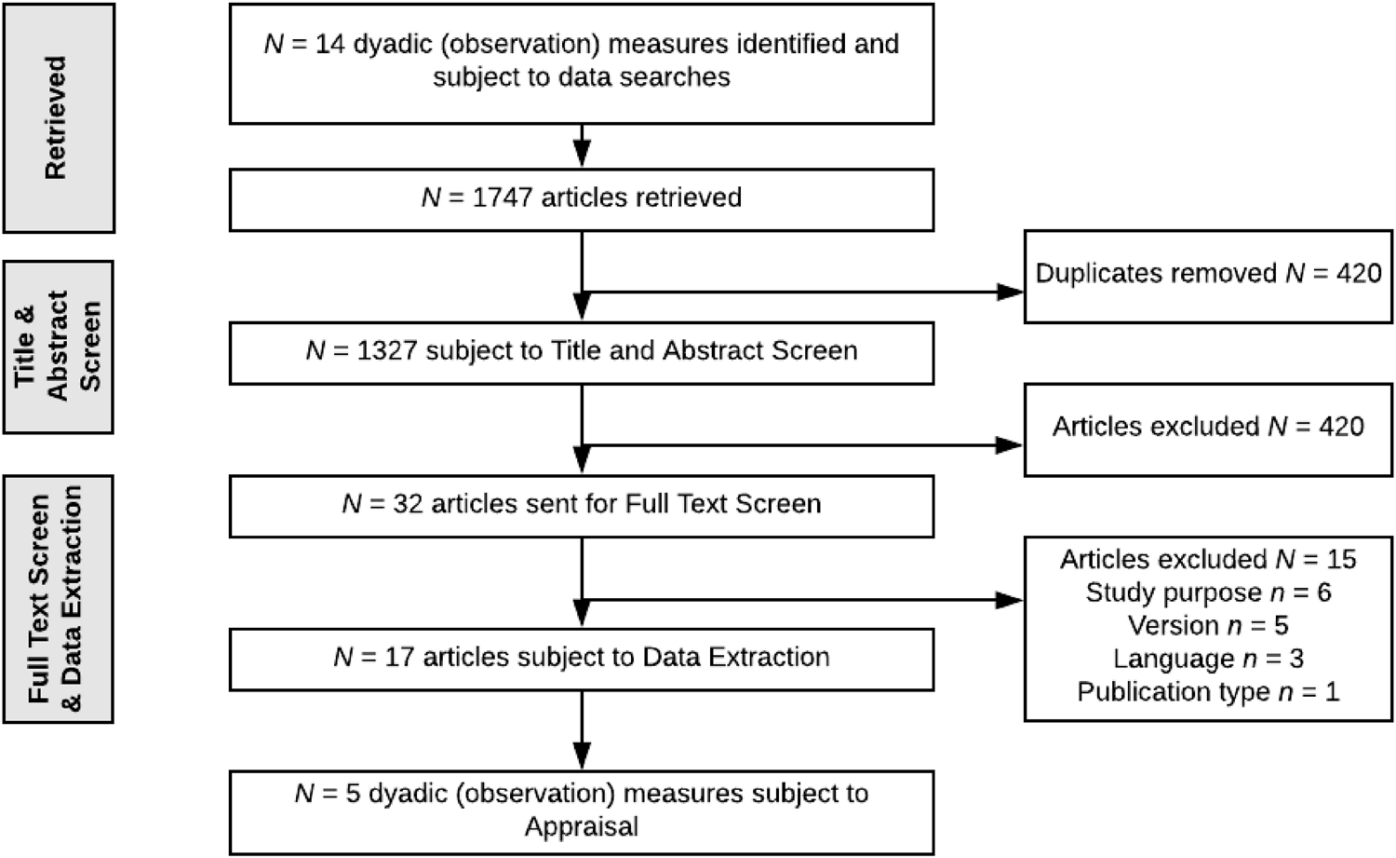
Flowchart of retrievals for Search 2, a systematic review of the psychometric properties of observation measures commonly used in RCTs of parenting programs

### Article Selection and Data Extraction

All retrieved articles were downloaded into an Endnote database and duplicates removed. Three authors (SB, NG, and ZH) independently performed a title and abstract screen of the remaining articles before performing a full-text screen applying the inclusion and exclusion criteria outlined above. Prior to data extraction inter-rater reliability checks were performed on a 20% random selection of all identified and included articles, and a 20% random selection of all excluded articles by two of the three authors. There were no recorded disagreements between authors.

Three authors (SB, NG, and KT) independently extracted data from the remaining articles using a google form to enable consistency. Data that were extracted were study authors, study design (i.e., parallel RCT or cluster), parenting program name and type (i.e., group or one-to-one), country of study, sample size and characteristics (i.e., age, gender, primary caregiver, ethnicity), the reported measures and their defined constructs according to our initial domain mapping exercise.

The data were then synthesized by two authors (SB and NG). This process sought to identify each individual measure and the number of times it occurred as an outcome in the included RCTs. The measures were then grouped within the domains [i.e., parent, child, dyadic by their format (i.e., questionnaires, developmental tests or observational tools)]. As the objective of Search 1 was to identify the most commonly reported measures used in RCT evaluations it was important that measures sent to Search 2 were widely used in the evaluation of parenting program research. To avoid bias that may occur by applying strict criteria the optimal threshold of appearances was explored. Across all three domains (parent, child, and dyadic outcomes) inclusion in at least three or more independent trials proved to be the optimum cut-off and subsequently this threshold was applied to identify the most relevant measures of interest.

### Search 2: Identifying the Development and Validation Studies of Eligible Measures

#### Eligibility Criteria

Dyadic measures identified in Search 1 were eligible if they were: (1) quantitative; (2) designed for the observation of the interaction between one parent and one infant by an external observer; (3) the latest version/edition; and (4) developed/administered/coded in the English language. Measures were excluded if they: (1) primarily measured constructs other than those defined in the initial domain mapping exercise; (2) were completed solely via parent-report; (3) had no full-text article available that either described or psychometrically evaluated the measure; and (4) had been developed/administered in another language i.e., not English.

For each measure identified in Search 1, two types of papers were considered for Search 2; those describing the development or application of the measures, and those evaluating the measures psychometric properties. Inclusion criteria were papers which: (1) described the development or evaluation of one of the identified eligible observational tools; (2) reported on a sample of expectant parents, mothers and/or fathers and other types of primary carer, of children up to and including the age of 5 years; (3) were published in the English language; and (4) was published in peer-reviewed scientific literature. Exclusion criteria for retrieved articles were the opposite of the above, in addition to: (1) the sample comprised exclusively of clinical sub-populations diagnosed with disorders unrelated to the objective of parenting programs (i.e., children with autism/cancer were excluded but adult populations with depression or children with social and emotional difficulties were not) and (2) the article did not provide sufficient information to determine eligibility.

### Search Strategy

To identify eligible articles for Search 2 a new database search which drew upon a complex key search term syntax developed by Terwee et al. (2009) and implemented by Bryant et al. (2014) and McConachie et al. (2015) for identifying studies on measurement properties was constructed. Five commercial platforms hosting the same databases used for Search 1 (with the exception of Cochrane, DARE, HTA and NHS EED) were searched systematically using the search strategy presented in Online Resource Table 1 in November 2016. Retrieved articles were then downloaded into an Endnote database and were subject to a title and abstract screen by two authors (NG and SB). Articles meeting the initial inclusion/exclusion criteria were then subject to a full-text screen to assess eligibility for data extraction by three of the authors (NG, SB and AD). Inter-rater reliability checks were performed on a 20% random selection of all identified and included articles retained for each tool, and a random 20% selection of all articles excluded during the full-text screen. Approximately, 1% of all papers resulted in a disagreement between two of the authors. Disagreements were resolved via consultation with the third reviewer who had not been involved in the initial screening or reliability check of that particular article.

**Table 1:** Scoring format and constructs of direct observational measures used in three or more RCT evaluations of parenting programs

Measure (citation)	Age range	Scales	Variables	Parent constructs	Child constructs	Dyadic constructs	Total items	Scoring format	Time (min)	Training/availability/cost
AQS Waters and Deane (1985)	1–5 years	7 Infant scales; 4 Criterion scores	Security dependency sociability social desirability	N/A	Proximity/exploration Differential responsiveness to caregiver Positive affect Sociability Independence Social perceptiveness Endurance Object use	N/A	90	Items scored as being ‘most-like, neutral, or unlike’ the child and are then sorted into three groups, or nine clusters. The nine clusters are scored from 1 to 9	60–120	Yes/no training information available/cost unknown
CSBS-DP behavior sample Wetherby and Prizant (2002)	6–24 months	7 Infant cluster scores 3 Composite scores 1 Total score	Social Speech Symbolic	N/A	Communicative function Communicative means (gestural) Communicative means (vocal) Communicative means (verbal) Reciprocity Social affective signaling Symbolic behavior	N/A	22	Clusters scored on Likert scale (0–5) Composite variable ranges Social 0–64 Speech 0–54 Symbolic 0–53	30–40	Yes/training provided via manual/full kit cost $399.00
EAS Biringen et al. (1998)	0–14 years	4 Caregiver scales 2 Infant scales 1 Total score 1 Global rating	N/A	Sensitivity Structuring Nonintrusiveness Nonhostility	Responsiveness to adults Involvement to adult	N/A	42	3-point or 7-point global rating (higher values indicate more optimal behavior)	20–30	Yes/ Training available online or in vivo (3–4 full days)/ Cost unknown
IT-HOME Caldwell and Bradley (1984)	0–3 years	6 Dyadic scales 1 Total score	N/A	N/A	N/A	Parental responsivity Acceptance of child Organization of the environment Learning materials Parental involvement Variety in experience	45	Yes/No Higher scores indicate greater interaction and physical environment	90	Yes/ Training provided via manual/ Approx. $100 for full kit
EC-HOME Caldwell and Bradley (1984)	3–6 years	8 Dyadic scales 1 Total score	N/A	N/A	N/A	Learning materials Language stimulation Physical environment Parental responsivity Learning stimulation Modeling of social maturity Variety in experience Acceptance of child	55	Yes/No Higher scores indicate greater interaction and physical environment	90	Yes/ Training provided via manual/ Approx. $100 for full kit

*AQS* Attachment Q-Sort, *CSBS-DP* Communication and Symbolic Behavior Scales-Developmental Profile, *EAS* Emotional Availability Scales, *EC-HOME* Early Childhood Home Observation Measurement of the Environment, *IT-HOME* Infant Home Observation Measurement of the Environment, *N*/*A* Not Applicable

### Data Extraction

Data were extracted from all eligible articles retrieved from Search 2 onto pre-determined data extraction forms using Qualtrics software. A systematic approach was taken to capture both the quality and evaluation of findings reported in eligible articles according to the structure of two sources: (1) the COSMIN (Terwee et al. 2011a) checklist, and (2) the Terwee et al. (2011b) quality criteria for measurement properties checklist (see http://www.cosmin.nl/ for further information).

To ensure that each of the included studies met the standards for good methodological quality, and that the risk of bias was minimal, the COSMIN was used as a measure of the article’s methodological quality. The COSMIN was developed via a Delphi study in response to the need for a standardized method to assess measurement studies and consistent application of psychometric definitions. The COSMIN was selected for the purposes of the current review over other checklists due to its advantages of facilitating comparisons between different measurement studies (Paiva et al. 2018). The COSMIN is applicable for both Classical Test Theory (CTT) and Item Response Theory (IRT) studies which are assessed according to 10-psychometric domains of interest, each with varying number of items: (1) internal consistency (11 items), (2) reliability (14 items), (3) measurement error (11 items), (4) content validity (5 items), (5) structural validity (7 items), (6) hypothesis testing (10 items), (7) cross-cultural validity (15 items), (8) criterion validity (7 items), (9) responsiveness (18 items), and (10) interpretability^1^ (7 items). Items across all 10-psychometric domains take into account both the design (missing items and sample size) and statistical reporting (specific analysis performed) of the study using a four-point scale (i.e., poor, fair, good, or excellent).

Applying the COSMIN taxonomy and definitions (de Vet et al. 2015; Terwee et al. 2007) three authors (NG, SB, and AD) independently extracted data from the eligible articles. Authors only extracted data relating to the specific psychometric domains reported in each study; no study was penalized for not reporting on all 10-psychometric domains. Each psychometric property reported in a given article was then provided an overall rating for its methodological quality based on COSMIN criteria of taking the lowest rating of any item within a domain i.e., worse score counts (Terwee et al. 2011a). Prior to data synthesis, inter-rater reliability checks were performed on 100% of the overall quality ratings. Two authors resolved disagreement through consensus. If no decision could be made the third authors was asked to make a final decision.

Following completion of the assessment of methodological quality using the COSMIN checklist, the quality of the psychometric evidence provided for each domain reported within each individual study was assessed using the Terwee et al. (2011b) checklist. This checklist mirrors the 10-domains captured by the COSMIN with findings across each domain rated on a three-point scale (positive, indeterminate, or negative). To ensure the checklist met the needs of the review some modifications were made to ensure definitions were transparent and easily applied across all of the included studies (see Online Resource Table 2). To ensure that we did not undermine the integrity of the results by modifying a standardized measure, the final criteria included a combination of the original (2007) definitions (where the criteria has not been recently amended), more recently updated guidelines (where the 2007 definition has been recently changed) and additional criteria implemented by recent users of the checklist (where definitions were previously obsolete).

**Table 2:** Summary of study characteristics

Journal author (date) Country	*N* Methods	Parent age in years (SD)	Child Age (SD)	% Female (parent)	% Female (child)	Predominant ethnicity (%)	Setting(s) in which the study was conducted	Recruitment methods	Location of observation	Task	Live or video
AQS											
Strayer et al. (1995) USA	67	NK	20–36 months	NK	50	NK	General Population Community Longitudinal study	Random	Home	Sorting procedure	Live
Tarabulsy et al. (1997) Canada	79	29 (4.9)	8 months	100	NK	NK	General population Community Hospital	Convenience	Home	Sorting procedure	Live
Teti and McGourty (1996) USA	40	31.67 (4.24)	31 months (14)	100	50	Caucasian (100)	General population Community Longitudinal study	Random	Home	Sorting procedure	Live
CSBS-DP Behavior Sample											
Chambers et al. (2016) South Africa	67	31.45 (5.06)	12–24 months	100	53	White (76)	General population Community	Convenience	Home Clinic Child-Care	CSBS-DP specific	Video
Eadie et al. (2010) Australia	728	NK	12 months (0.3)	NK	49.5	NK	General population Community Longitudinal study	Convenience	Home	CSBS-DP specific	Live Video
Watt et al. (2006) USA	160	Mothers 31.90 (5.25) Fathers 34.94 (5.99)	Early sample 14.31 months (1.36) Late sample 19.67 months (1.16)	50	43	Caucasian (80)	General population Community Cohort study	Convenience	NK	CSBS-DP specific	Video
Wetherby et al. (2002) USA	364	Mothers 31 (6) Fathers 34 (6.9)	18 (3.6)	NK	41.6	Caucasian (76.7)	General population Community Cohort study	Random	Clinic	CSBS-DP specific	Video
EAS											
Biringen et al. (2005) USA	Study 1: 36 Study 2: 57	NK	Study 1: 12 months Study 2: 4–5 years	100	NK	NK	General population Community	Convenience	Home	Naturalistic	Live
Bornstein et al. (2006a) USA	34	31.76 (2.19)	23.91 months (0.46)	100	50	White (100)	General population Community	Convenience	Home	Naturalistic	Video
Bornstein et al. (2006b) USA	52	30.09 (4.87)	161.8 d (4.4)	100	56	European American (100)	General population Community	Convenience	Laboratory	Naturalistic	Video
EC-HOME											
Sugland et al. (1995) USA	819	NK	36 months	100	NK	African American (53)	General population Community Cohort study	Convenience	Home	Semi-structured	Live
IT-HOME											
Linver et al. (2004) USA	2409	NK	5.1–12 months	100	49–53	European American (20–84)	General population Community Cohort study	Random Convenience	Home	Semi-structured	Live
Mitchell and Gray (1981) USA	144	NK	4–12 months	100	NK	NK	General population Community Hospital Cohort study	Convenience	Home Clinic	Semi-structured	Live
Stevens and Bakeman (1985) USA	213	22.9	13–30 months	100	NK	African American (67)	General population Community	Convenience	Home	Semi-structured	Live
Tesh and Holditch-Davis (1997) USA	53	30.2 (6.5)	3 years	100	47.2	African American (50.9)	Hospital Primary Care	Convenience	Home	Naturalistic	Live
IT and EC-HOME											
Bradley et al. (1994) USA	870	23.1–27.2	1–3 years	100	NK	African American (52.9)	General population Community Cohort study	Random Convenience	Home	Naturalistic	Live
Mundfrom et al. (1993) USA	900	24.8 (6.03)	11.5–37 months	100	NK	African American (53)	General population Community Cohort study	Random Convenience	Home	Naturalistic	Live

*AQS* Attachment Q-Sort, *CSBS-DP* Communication and Symbolic Behavior Scales-Developmental Profile, *EAS* Emotional Availability Scales, *EC-HOME* Early Childhood Home Observation Measurement of the Environment, *IT-HOME* Infant Home Observation Measurement of the Environment, *NK* Not known

### Data Synthesis

To provide an overall evaluation of each measures reported level of evidence across the 10-psychometric domains three authors (NG, SB, and AD) pooled the methodological quality ratings (i.e., poor, fair, good, or excellent) with the ratings applied for their reported psychometric evidence [i.e., positive (+), indefinite (?), or negative (−) ratings]. To ensure that no measure was unfairly disadvantaged during the data synthesis stage the following rules were applied to account for differences in the number of studies providing supporting evidence for each of the 10-psychometric domains;

#### Strong Level of Evidence (+++ or −−−)

This rating was applied when the evidence for the target psychometric property of a measure was supported by consistently positive or negative findings in multiple studies (two or more) rated good in methodological quality, or in one study of excellent methodology quality.

#### Moderate Level of Evidence (++ or −−)

This rating was applied when the evidence for the target psychometric property of a measure was supported by consistently positive or negative findings in multiple studies (two or more) rated fair in methodological quality, or in one study of good methodological quality.

#### Limited Level of Evidence (+ or −)

This rating was applied when the evidence for the target psychometric property of a measure was supported by positive or negative findings from one study rated fair in methodological quality.

#### Conflicting Level of Evidence (+/)

This rating was applied when the evidence for the target psychometric property of a measure was supported by studies of a similar quality with conflicting findings.

#### Unknown (?)

This rating was applied when the evidence for the target psychometric property of a measure was supported only by studies of poor methodological quality or the criteria was not met for a positive or negative rating in the majority of reviewed studies.

## Results

A total of 16,761 articles were retrieved in Search 1, with 279 articles progressing to the data extraction stage (see Online Resource Fig. 1). The 279 articles comprised peer-reviewed and published RCT evaluations of 113 parenting programs delivered within clinics or communities as one-to-one or group-based programs. Sample characteristics reported across individual studies varied in terms of size (range *N* = 24 to 5563), target caregiver (e.g., mothers only, or mothers and fathers), ethnicity and country of study, suggesting a full representation of the available literature. A total of 480 measures were reported across the 279 studies including questionnaires (*N* = 268), developmental tests (*N* = 55), observational tools (*N* = 106), and other formats (*N* = 51) such as clinical interview schedules. Assessment of the varying frequencies of use/occurrence of measures across independent RCTs (≥ 1, ≥ 2, ≥ 3, ≥ 4) was conducted to determine the optimal criteria that best represented the term ‘commonly used’. Application of these thresholds across all three domains indicated that ≥ 1 and ≥ 2, yielded too many measures for the review to be manageable and meaningful, whilst the difference between the ≥ 3 and ≥ 4 criteria was minimal. Subsequently, three or more appearances was deemed appropriate for all domains and this criterion was applied leaving 14 dyadic outcome measures (all observational tools) eligible for progression to Search 2 (Online Resource Table 3).

**Table 3:** A summary of the overall quality of the psychometric measurement for each of the five reviewed measures based on the synthesized evidence of the 17 articles reviewed

Measure (total number of studies reviewed)	Internal consistency	Test–re-test reliability	Inter-rater reliability	Structural validity	Convergent/divergent validity
AQS (3)	?		−−	+	−−
CSBS-DP behavior sample (4)	+++	++	++	?	
EAS (3)	?	−−	++		
IT-HOME (6)	−−			++	−−
EC-HOME (3)	−−			++	−

Strong level of evidence (+++ or −−−)/moderate level of evidence (++ or −−)/limited level of evidence (+ or −)/conflicting level of evidence (+/)/unknown (?)

*AQS* Attachment Q-Sort, *CSBS-DP* Communication and Symbolic Behavior Scales-Developmental Profile, *EAS* Emotional Availability Scales, *EC-HOME* Early Childhood Home Observation Measurement of the Environment, *IT-HOME* Infant Home Observation Measurement of the Environment, *NK* Not known

Search 2 yielded a total of 1747 articles describing the development and/or validation of the 14 observational measures identified in Search 1. Each of these articles were retrieved and assessed against the inclusion/exclusion criteria by three authors (NG, SB, and AD). Of those articles retrieved 420 duplicates were removed (Fig. 1). An initial title and abstract screen excluded 1295 articles and the full-text screen a further 15 articles. This left 17 articles for inclusion in the final review.

These 17 articles described the development/validation of only five of the original 14 observational measures (Table 1). Validation papers were available for the nine measures which were not carried forward. Table 2 provides a summary of the sample characteristics of the 17 studies providing evidence for the psychometric properties of the five observational measures. Table 3 provides the summary overview of the level of evidence for each of the psychometric domains reported for the five observational measures as rated used the COSMIN (Terwee et al. 2011a) and modified Terwee checklist (2011b). A summary of each studies findings are available in Online Resources Table 4.

### Attachment Q-Sort (AQS; Waters and Deane 1985)

The current review identified three studies, which presented psychometric information for the AQS (Strayer et al. 1995; Tarabulsy et al. 1997; Teti and McGourty 1996). Using evidence drawn from the three supporting studies the AQS demonstrates an unknown level of internal consistency, negative evidence for inter-rater reliability, limited positive evidence for structural validity and negative evidence for convergent validity. Subsequently, when rated using the COSMIN and Terwee checklists (2011a, b) these findings suggest little psychometric evidence to support the use of the AQS in an English-speaking sample of children aged from 8 to 36 months.

### Communication and Symbolic Behavior Scales-Developmental Profile Behavior Sample (CSBS-DP; Wetherby and Prizant 2002)

A total of four studies were identified to provide evidence for the psychometric properties of the CSBS-DP behavior sample (Chambers et al. 2016; Eadie et al. 2010; Watt et al. 2006; Wetherby et al. 2002). Evidence drawn from the four reviewed studies suggest that the CSBS-DP behavior sample has strong evidence for internal consistency at the cluster, composite and total score level, a moderate level of positive ratings for test–re-test reliability over a 4-month period, a moderate level of positive evidence for inter-rater reliability, and an unknown estimate for its structural validity using the three-factor model. Subsequently, when rated using the COSMIN and Terwee et al. checklists (2011a, b) these findings suggest good psychometric evidence to support the use of the CSBS-DP in an English-speaking sample of children aged from 12 to 24 months.

### Emotional Availability Scales (EAS; Birigen et al. 1998)

Three studies were identified which reported on the psychometric properties of the EAS for the target population (Biringen et al. 2005; Bornstein et al. 2006a, b). The combined evidence for the EAS is inconclusive. The true estimate for its internal consistency is unknown due to the reviewed study being rated as poor in methodological quality. Evidence supporting its test–re-test reliability is negative, whilst inter-rater reliability indicates a moderate level of positive evidence according to Terwee standards (2011b). Subsequently, these findings suggest little psychometric evidence to support the use of the EAS in an English-speaking sample of children aged from 5 months to 5 years.

### Infant-Toddler Home Observational Measurement of the Environment (IT-HOME; Caldwell and Bradley 1984)

Six papers were eligible and included in the current review, which reported on the psychometric properties of the IT-HOME (Bradley et al. 1994; Linver et al. 2004; Mitchell and Gray 1981; Mundfrom et al. 1993; Stevens and Bakeman 1985; Tesh and Holditch-Davies 1997). The evidence to support the psychometric utility of IT-HOME for English-speaking samples of children aged from birth to 3 years indicates conflicting evidence for internal consistency and moderate positive evidence for its structural validity using a six-factor solution. In addition, the evidence to support the convergent/divergent property of the IT-HOME is currently inconclusive according to Terwee et al. standards (2011b). Subsequently, these findings suggest little psychometric evidence to support the use of the IT-HOME with a population of children aged 4 to 36 months.

### Early Childhood Home Observational Measure of the Environment (EC-HOME; Caldwell and Bradley 1984)

A total of three papers were eligible for inclusion in the second stage review, which provided evidence to support the psychometric properties of the EC-HOME (Bradley et al. 1994; Mundfrom et al. 1993; Sugland et al. 1995). To sum, according to Terwee standards, evidence for the EC-HOME was moderately negative for internal consistency and convergent validity, and moderately positive for its structural validity. Subsequently, when rated using the COSMIN and Terwee et al. checklists (2011a, b) these findings suggest little psychometric evidence to support the use of the EC-HOME in an English-speaking sample of children aged from 11 to 37 months.

## Discussion

The purpose of the current review was to identify commonly used observational measures reported as part of RCT evaluations of parenting programs (designed for parents of children aged up to and including 5 years), and to then synthesize the current psychometric evidence for these measures with a view to make recommendations for use in further research (i.e., other RCTs and service evaluations) and clinical practice. We did not stipulate a specific aspect of parent–child interaction, nor any particular measure that we were most interested in assessing in order to ensure that we identified a broad range of different constructs being assessed. It is recognized that the final batch of measures include scales that are not directly related to parent–child interactions i.e., the IT- and EC-HOME, however we included a full review of the measure to permit an assessment of structural validity. Five observational measures were identified with 17 articles retrieved that provided supporting evidence of the development or validation of these measures with an English-speaking sample. Of those measures identified and evaluated, the CSBS-DP behavior sample, a macro observational measure of children’s social communication development, was shown to have the strongest evidence to support its psychometric reliability and validity. Although, two of the four reviewed studies were not conducted independently of the developers. Overall, the methodological quality of all studies supporting the development or validation of the five measures was rated poor according to COSMIN and Terwee checklists (Terwee et al. 2011a, b) due in part to the small sample sizes and poor study design. Moreover, the evidence provided to support the five measures predominantly spanned the birth to three age range, with little or no evidence for the measure’s suitability for use with 3–5 year old’s. Consequently, it is not possible to confidently state if the five observational measures included in the review are valid and reliable for use with our target population (0–5 years).

The most striking finding from this review is the lack of evidence across the range of components of validity for the five included measures. Not one of the 17 supporting articles reported on the content, criterion, or cross-cultural validity of the five measures under review. More surprisingly, none of the included articles reported on responsivity (i.e., stability or sensitivity to change) despite these tools being used, although not originally designed, for the purposes of evaluating change following intervention. Previous researchers (Lotzin et al. 2015; Munson and Odom 1996) have suggested that users of observational scales should first look to the validity estimates of the measure, before looking at reliability estimates and other observation specific considerations (i.e., task, setting etc.). However, the lack of evidence to support four of the five aspects of validity, in addition to the lack of evidence for sensitivity to change suggests that this is still problematic. Our review highlights the continuing need for further work so that researchers and practitioners can be confident when selecting measures that have real world implications for assessment, evaluation and monitoring change over time.

Internal consistency, structural validity and inter-rater reliability were the most common psychometric properties for all five measures. These findings support the conclusions drawn from previous systematic reviews which highlight the ease with which internal consistency estimates can be made for those measures which lend themselves to this psychometric property i.e., non-dichotomous scales (Aspland and Gardner 2003; Lotzin et al. 2015). Evidence from the CSBS-DP behavior sample measure proved to have the strongest evidence for internal consistency, meeting the COSMIN (Terwee et al. 2011a, b) criteria at both the composite and total score level. Structural validity was the second most commonly reported psychometric property with both the IT-HOME (birth to 3 years) and EC-HOME (3–6 years) proving the strongest measures within this category. Overall, the findings seem to suggest that further examination of the structural validity of complex observational measures is needed at all levels of item analyses (i.e., composite, cluster, total scores) to ensure that they meet the necessary specific statistical standards.

Inter-rater reliability estimates were reported for three of the five measures, with only the IT-HOME and EC-HOME not having any supporting evidence for this property possibly because some of the items are parent reported. The CSBS-DP behavior sample measure and the EAS both demonstrated moderate levels of evidence for achieving the COSMIN standard level of inter-rater agreement, however the AQS did not. These findings are concerning given that three of the five measures reviewed here could be considered complex coding schemes (AQS; CSBS-DP behavior sample and EAS). As a result, they require substantial time and cost to train users to become competent and carry out and conduct the coding of the interaction (see Online Resources Table 4). It could be argued that COSMIN thresholds for inter-rater reliability are restrictive and do not lend themselves readily to observational methods where acceptable levels of agreement can be as low as .61 (see Landis and Koch 1977). However, poor levels of inter-rater reliability fundamentally undermine the validity of the data generated and given the need for replicability in observational assessments achieving high levels of inter-rater reliability ensures that data can be relied upon to aid practitioners in making informed decisions regarding referrals for treatment, and researchers in making valid judgements on outcomes following program attendance (Yoder and Symons 2010).

This review is the first to investigate the psychometric evidence to support the use of commonly used observational measures adopted as outcome measures as part of RCT evaluations of parenting programs. Observational assessments are increasingly being adopted as part of research as outcome measures to monitor change over time following intervention and the findings indicate that many aspects of parent–child interaction are being assessed as part of RCT evaluations (attachment, emotional availability, communication, and home environments). Of the five reviewed observational assessments, two are also known to be used in clinical practice to screen and signpost parents to programs in routine service delivery i.e., the AQS and the EAS. Consequently, it is becoming increasingly important that such measures are routinely assessed for their level of validity, reliability and responsiveness. We selected measures commonly used in RCTs (irrespective of the unit of detail i.e., micro or macro, or concept of interest) as we assumed these would be the most robust measures available and most likely to be used in practice. However, the findings from this review highlight that despite their widespread adoption within research, further work is required to ensure that they consistently meet the statistical standards for reliability and validity with this young age group before being used in routine practice.

To address some of the limitations identified in previous research, namely inconsistencies in the synthesis and rating of methodological and psychometric evidence, we adopted both the COSMIN (Terwee et al. 2011a), a measure of methodological quality, and the Terwee checklist, a measure for assessing measurement properties (Terwee et al. 2011b). The decision to use these two tools was pragmatic. Firstly, the COSMIN and Terwee checklists are being adopted in the medical literature as a standard process for extracting and synthesizing data for systematic reviews focusing on measurement properties. Within the social sciences such standard processes do not exist. Secondly, both tools work in tandem and it was hoped that this would ensure a standardized approach to strengthen the current review’s interpretability, generalizability and replicability for future efforts in this area. Despite these strengths, due to its foundations origin within the medical literature, the checklists proved unhelpful in some instances when trying to make concrete decisions about the overall psychometric quality of measures. Consequently, whilst these tools are now been being adopted across a variety of fields to assess a host of measures, including the observation of early child behavior and dyadic processes (McConachie et al. 2015), further work is needed to: (1) refine the language and increase clarity of instructions, (2) understand whether the current thresholds are appropriate across the board or should be lowered when applied to specific measures i.e., observational measures, and (3) make them accessible to all users irrespective of the type of data/measures they are working with.

This review focused only on those measures reported in three or more RCTs identified in Search 1, applying strict criteria about our population of interest. As a result, some measures that we identified in Search 1 were not included in the final review e.g., the Strange Situation Procedure (Ainsworth 1977), and the final list may not represent those commonly used as assessment measures in clinical practice. The obvious exclusion of some well-known measures used for evaluating parenting programs designed for older children, e.g., the Dyadic Parent–Child Interaction Coding System (DPICS: Eyberg and Robinson 1983), and some measures used routinely in clinical practice (i.e., Strange Situation Procedure) that were originally identified in Search 1 is acknowledged to be a limitation of the current review. Despite this, we conducted a thorough assessment to identify the impact of applying various thresholds to the list of measures identified in Search 1, and occurrence in three or more RCTs was shown to best represent the term ‘commonly used’. Whilst previous reviews have assessed the psychometric evidence for some of the measures not reviewed here i.e., measures not used in RCTs, there is a need for future research to pull this information together in one format to facilitate access, reduce time inefficiencies when searching for such information, and to ensure that researchers and practitioners are consistently adopting robust measures for assessment and to measure change.

A further limitation of the current review is the application of language restrictions. The decision to exclude non-English publications was a pragmatic one made at the inception of the review due to the costs required to translate articles. The decision to exclude different language versions of a measure, even if reported in English, was also pragmatic and was made at the conclusion of Search 1 due to the quantity of articles retrieved during our initial database searches. Whilst English is often considered the universal language of science we acknowledge that language restrictions in systematic reviews can result in a biased representation of the literature (Grégoire et al. 1995; Morrison et al. 2012; Wang et al. 2015). Consequently, our findings should not be regarded as conclusive evidence of the reviewed measure’s reliability and validity, and we advise future researchers include the results drawn from non-English publications in subsequent review updates.

The overarching aim of this review was to identify commonly used measures in RCTs that measure some aspect of parent–child interaction in order to recommend a small battery of measures that could be used by both researchers and practitioners. Assessment of parent–child interactions is an important feature of child health and wellbeing provision, allowing early challenges to be recognized and appropriate help to be mobilized (Axford et al. 2015). Practitioners and researchers are committed to assessing dyadic interactions (Appleton et al. 2012); however, skills in detailed assessment of parent-infant interactions do not necessarily correlate with years of professional experience. It seems likely that additional post qualification training is required to perform accurate observational assessments (Appleton et al. 2012; Kristensen et al. 2017). In terms of clinical practice, an absence of financial resource and thus an absence of additional training in the use of observational measures could mean that problematic parent–child interactions are being, unintentionally, underestimated. This means that services adopting parenting training programs need to take a comprehensive approach to service provision by training workforces making referrals to parenting programs to assess dyadic interactions. Training for referrers and researchers should address knowledge of parent–child interactions and skill gaps in the application of validated observational measures for making assessments and assessing outcomes/change over time. Such training would ensure that those parents identified as eligible for additional support stand most to benefit from the parenting intervention thus ensuring that money is allocated to good measures that validly and reliably assess need for services. What is more, practitioners trained in dyadic interaction assessment can repeat their observation as part of ongoing support and strategies for individual families participating in parenting programs, thereby ensuring adequate return of social investment.

Previously, Lotzin et al. (2015) suggested that the quality of development and validation studies for observational measures across the board needs improving, and measures need further validation. The current review contributes to the existing body of knowledge on parenting support, by drilling down and examining the quality of one feature (observational tools used) of those studies (RCTs) conducted in this field. By clarifying the quality of the measures used within ‘existing research’ there is an opportunity to differentiate further between the range of evidence available. The scarcity of high-quality psychometric evidence to support the five observational measures of parent–child interaction identified within this review as being used in parenting research highlights the need for further examination of these measures. We cannot be confident from the findings of this review in recommending one of these measures over another for the purposes of screening or assessing outcomes/change in parent–child interaction as part of routine practice or research studies. In addition, due to the few studies which reported psychometric properties spanning the entirety of our target population (birth to five) we highly recommend further validation of these measures across the age range before applying them as outcome measures within effectiveness trials or continuing their use within clinical practice.

## Electronic supplementary material

Below is the link to the electronic supplementary material.

Supplementary material 1 (PDF 94 KB)

Supplementary material 2 (PDF 100 KB)

Supplementary material 3 (PDF 85 KB)

Supplementary material 4 (PDF 155 KB)

## Footnotes

1 The items relating to interpretability are extracted solely for the purposes of study description and do not contribute to a measures overall quality rating.
