# Supplementary material for: Psychometric Properties of Parent–Child (0–5 years) Interaction Outcome Measures as Used in Randomized Controlled Trials of Parent Programs: A Systematic Review
Source: Clin Child Fam Psychol Rev. 2019 Feb 7;22(2):253–71. doi: 10.1007/s10567-019-00275-3 (PMC6478772; doi:10.1007/s10567-019-00275-3)
Supplement: Supplementary file 1 — Supplementary material 1 (PDF 94 KB) [file 10567_2019_275_MOESM1_ESM.pdf]

Psychometric Properties of Parent-Child (0-5 years) Interaction Outcome Measures as used in Randomized Controlled Trials of Parent Programs: A Systematic Review

Nicole Gridley, Sarah Blower, Abby Dunn, and Tracey Bywater

Department of Health Sciences, University of York

Karen Whittaker

School of Nursing, University of Central Lancashire

Maria Bryant

Leeds Institute of Clinical Trials Research, University of Leeds

Correspondence concerning this article should be addressed to Nicole Gridley, School of Education, Leeds Beckett University, Leeds, LS6 3QQ
Email: n.gridley@leedsbeckett.ac.uk

Online Resources Table 1.

Example of search syntax used to identify eligible articles

Search Term
(Emotional Availability Scales) AND (instrumentation.sh. OR methods.sh. OR Validation Studies.pt. OR Comparative Study.pt. OR psychometrics/ OR psychometry*.ab,ti. OR clinometer*.tw. OR clinometer*.tw. OR Outcome Assessment OR outcome assessment.ab,ti. OR outcome measure*.tw. OR observer variation/ OR observer variation.ab,ti. OR Health Status Indicators/ OR reproducibility of results/ OR reproducible*.ab,ti. OR discriminant analysis/ OR reliab*.ab,ti. OR unreliable*.ab,ti. OR valid*.ab,ti. OR coefficient.ab,ti. OR homogeneity.ab,ti. OR homogeneous.ab,ti. OR internal consistency.ab,ti. OR (cronbach*.ab,ti. AND (alpha.ab,ti. OR alphas.ab,ti.)) OR (item.ab,ti. AND (correlation*.ab,ti. OR selection*.ab,ti. OR reduction*.ab,ti.)) OR agreement.ab,ti. OR precision.ab,ti. OR imprecision.ab,ti. OR precise values.ab,ti. OR test-retest.ab,ti. OR (test.ab,ti. AND retest.ab,ti.) OR (reliab*.ab,ti. AND (test.ab,ti. OR retest.ab,ti.)) OR stability.ab,ti. OR interrater.ab,ti. OR interpreter.ab,ti. OR intrarater.ab,ti. OR intra-rater.ab,ti. OR intertester.ab,ti. OR inter-tester.ab,ti. OR intratester.ab,ti. OR intra-tester.ab,ti. OR interobserver.ab,ti. OR inter-observer.ab,ti. OR intraobserver.ab,ti. OR intraobserver.ab,ti. OR intertechnician.ab,ti. OR inter-technician.ab,ti. OR intratechnician.ab,ti. OR intra-technician.ab,ti. OR interexaminer.ab,ti. OR inter-examiner.ab,ti. OR intraexaminer.ab,ti. OR intraexaminer. ab,ti. OR interassay.ab,ti. OR inter-assay.ab,ti. OR intraassay.ab,ti. OR intra-assay.ab,ti. OR interindividual.ab,ti. OR inter-individual.ab,ti. OR intraindividual.ab,ti. OR intra-individual.ab,ti. OR interparticipant.ab,ti. OR inter-participant.ab,ti. OR intraparticipant.ab,ti. OR intra-participant.ab,ti. OR kappa.ab,ti. OR kappas.ab,ti. OR repeatable*.ab,ti. OR ((replica*.ab,ti. OR repeated.ab,ti.) AND (measure.ab,ti. OR measures.ab,ti. OR findings.ab,ti. OR result.ab,ti. OR results.ab,ti. OR test.ab,ti. OR tests.ab,ti.)) OR generalize*.ab,ti. OR generalist*.ab,ti. OR concordance.ab,ti. OR (intraclass.ab,ti. AND correlation*.ab,ti.) OR discriminative.ab,ti. OR known group.ab,ti. OR factor analysis.ab,ti. OR factor analyses.ab,ti. OR dimension*.ab,ti. OR subscale*.ab,ti. OR (multitrait.ab,ti. AND scaling.ab,ti. AND (analysis.ab,ti. OR analyses.ab,ti.)) OR item discriminant.ab,ti. OR interscale correlation*.ab,ti. OR error.ab,ti. OR errors.ab,ti. OR individual variability.ab,ti. OR (variability.ab,ti. AND (analysis.ab,ti. OR values.ab,ti.)) OR (uncertainty.ab,ti. AND (measurement.ab,ti. OR measuring.ab,ti.)) OR standard error of measurement.ab,ti. OR sensitive*.ab,ti. OR responsive*.ab,ti. OR ((minimal.ab,ti. OR minimally.ab,ti. OR clinical.ab,ti. OR clinically.ab,ti.) AND (important.ab,ti. OR significant.ab,ti. OR detectable.ab,ti.) AND (change.ab,ti. OR difference.ab,ti.)) OR (small*.ab,ti. AND (real.ab,ti. OR detectable.ab,ti.) AND (change.ab,ti. OR difference.ab,ti.)) OR meaningful change.ab,ti. OR ceiling effect.ab,ti. OR floor effect.ab,ti. OR Item response model.ab,ti. OR IRT.ab,ti. OR rausch.ab,ti. OR Differential item functioning.ab,ti. OR DIF.ab,ti. OR computer adaptive testing.ab,ti. OR item bank.ab,ti. OR cross-cultural equivalence.ab,ti.)
