# Supplementary material for: Psychometric Properties of Parent–Child (0–5 years) Interaction Outcome Measures as Used in Randomized Controlled Trials of Parent Programs: A Systematic Review
Source: Clin Child Fam Psychol Rev. 2019 Feb 7;22(2):253–71. doi: 10.1007/s10567-019-00275-3 (PMC6478772; doi:10.1007/s10567-019-00275-3)
Supplement: Supplementary file 2 — Supplementary material 2 (PDF 100 KB) [file 10567_2019_275_MOESM2_ESM.pdf]

Psychometric Properties of Parent-Child (0-5 years) Interaction Outcome Measures as used in Randomized Controlled Trials of Parent Programs: A Systematic Review

Nicole Gridley, Sarah Blower, Abby Dunn, and Tracey Bywater

Department of Health Sciences, University of York

Karen Whittaker

School of Nursing, University of Central Lancashire

Maria Bryant

Leeds Institute of Clinical Trials Research, University of Leeds

Correspondence concerning this article should be addressed to Nicole Gridley, School of Education, Leeds Beckett University, Leeds, LS6 3QQ

Email: n.gridley@leedsbeckett.ac.uk

Online Resources Table 2.
Terwee (2011) criteria for good measurement properties

Measurement property	Rating	Criteria	Source
Reliability			
Internal consistency (CTT methods)	+	Cronbach alpha(s) > 0.70	Terwee et al. 2007
	?	Cronbach alpha(s) not determined OR dimensionality unknown OR conflicting evidence	
	-	Cronbach alpha(s) < 0.70	
Internal consistency (IRT methods)	+	Person Separation Index > 0.70	Heinl et al. (2016)
	?	Person Separation Index not determined	
	-	Person Separation Index <0.70	
Measurement error	+	MIC > SDC OR MIC outside the LOA	Terwee et al. (2007)
	?	MIC not defined	
	-	MIC < SDC OR MIC equals or inside LOA	
Reliability (inter-rater and test-retest)	+	ICC/weighted Kappa > 0.70 OR Pearson's r > 0.80	Heinl et al. (2016)
	?	Neither ICC/weighted Kappa or Pearson's r calculated	
	-	ICC/weighted Kappa > 0.70 OR Pearson's r > 0.80	
Validity			
Content validity	+	All items are considered to be relevant for the construct to be measured, for the target population, and for the purpose of the measurement AND the questionnaire is considered to be comprehensive	Terwee et al. (2007)
	?	Not enough information available	
	-	Not all items are considered to be relevant for the construct to be measured, for the target population, and for the purpose of the	

Measurement property	Rating	Criteria	Source
		measurement OR the questionnaire is not considered to be comprehensive	
Structural validity (CTT methods)	+	Factors should explain at least 50% of the variance OR CFI or TLI or comparable measure > 0.95 AND (RMSEA < 0.06 OR SRMR < 0.08)	Terwee et al. (2007) and modified version available on COSMIN website.
	?	Not all information for '+' reported	
	-	Criteria for '+' not met	
Structural validity (IRT methods)	+	Residual correlations among the items after controlling for the dominant factor < 0.20 OR Q3's < 0/37, item scalability > 0.30, IRT model fit: G2 > 0.01, no DIF for important subject characteristics (such as age, gender, education): McFadden's R2 < 0/02, OR no non-uniform DIF	Terwee et al. (2007) and modified version available on COSMIN website.
	?	Important statistics not reported	
	-	Criteria for '+' not met	
Hypothesis Testing (convergent/divergent validity)	+	Correlations with instruments measuring the same construct > 0.50 OR at least 75% of the results are in accordance with the hypotheses AND correlation with related constructs is higher than with unrelated constructs	Terwee et al. (2007)
	?	Solely correlations determined with unrelated constructs	
	-	Correlations with instruments measuring the same construct <0.50 OR < 75% of the results are in accordance with the hypotheses OR correlation with related constructs is higher than with unrelated constructs	
Hypothesis Testing (discriminant/known groups)	+	Differences in scores on the measurement instrument for all evaluated patient subgroups are statistically significant OR > 75% of results in accordance with hypotheses	Heinl et al. (2016)
	?	Some differences statistically significant, others not	

Measurement property	Rating	Criteria	Source
	-	Differences in scores on the measurement instrument for all evaluated patient subgroups are not statistically significant OR < 75% of results in accordance with hypotheses	
Criterion validity	+	Convincing arguments that gold standard is "gold" AND correlation with gold standard > 0.70 OR (sensitivity AND specificity > 70%)	Terwee et al. (2007) de Vet et al. (2015)
	?	Not all information for '+' reported	
	-	Criteria for '+' not met	
Responsiveness	+	SDC or SDC < MIC OR MIC outside the LOA OR RR > 1.96 OR AUC > .70	Modified Terwee et al. (2007)
	?	Doubtful design or method	
	-	SDC or SDC > MIC OR MIC equals or inside LOA OR RR < 1.96 OR AUC < .70 despite adequate design and methods	
