# Supplementary material for: Psychometric Properties of Parent–Child (0–5 years) Interaction Outcome Measures as Used in Randomized Controlled Trials of Parent Programs: A Systematic Review
Source: Clin Child Fam Psychol Rev. 2019 Feb 7;22(2):253–71. doi: 10.1007/s10567-019-00275-3 (PMC6478772; doi:10.1007/s10567-019-00275-3)
Supplement: Supplementary file 3 — Supplementary material 3 (PDF 85 KB) [file 10567_2019_275_MOESM3_ESM.pdf]

Psychometric Properties of Parent-Child (0-5 years) Interaction Outcome Measures as used in Randomized Controlled Trials of Parent Programs: A Systematic Review

Nicole Gridley, Sarah Blower, Abby Dunn, and Tracey Bywater

Department of Health Sciences, University of York

Karen Whittaker

School of Nursing, University of Central Lancashire

Maria Bryant

Leeds Institute of Clinical Trials Research, University of Leeds

Correspondence concerning this article should be addressed to Nicole Gridley, School of Education, Leeds Beckett University, Leeds, LS6 3QQ

Email: n.gridley@leedsbeckett.ac.uk

Online Resources Table 3.

List of all observational tools identified in Search 1 and thus carried forward for Search 2

Tool Name	Search 1 No of RCTs	Search 1 Articles retrieved	Search 2 Full Text Screen	Search 2 Excluded	Reason(s) for exclusion*
Ainsworth Maternal Sensitivity Scale	3	62	1	1	1
Attachment Q-Sort	4	149	7	4	1, 2, 3
Coders Impression Inventory	7	44	0	-	-
Communication Symbolic & Behaviour Scale	4	117	4	0	-
Dyadic Parent-Child Interaction Coding System	36	81	2	2	4, 5
Emotional Availability Scale	7	254	5	2	1, 3
Erikson's Rating Scale	4	52	0	-	-
Family Observation Schedule	6	84	0	-	-
Global Impressions of Parent-Child Interactions	4	57	0	-	-
HOME Inventory	25	675	10	3	6
Maternal Behavior Q-Sort	4	19	0	-	-
NICHD Study of Early Child Care & Youth Development	4	54	0	-	-
NCAST Parent-Child Interaction Scales	11	59	0	-	-
Relationship Process Code	4	122	0	-	-

*1 = Type of study/ 2 = Version/ 3 = Language of publication/ 4 = Publication type/ 5 = Age of child/ 6 = No access to full text
