# Supplementary material for: Psychometric Properties of Parent–Child (0–5 years) Interaction Outcome Measures as Used in Randomized Controlled Trials of Parent Programs: A Systematic Review
Source: Clin Child Fam Psychol Rev. 2019 Feb 7;22(2):253–71. doi: 10.1007/s10567-019-00275-3 (PMC6478772; doi:10.1007/s10567-019-00275-3)
Supplement: Supplementary file 4 — Supplementary material 4 (PDF 155 KB) [file 10567_2019_275_MOESM4_ESM.pdf]

Psychometric Properties of Parent-Child (0-5 years) Interaction Outcome Measures as used in Randomized Controlled Trials of Parent Programs: A Systematic Review

Nicole Gridley, Sarah Blower, Abby Dunn, and Tracey Bywater

Department of Health Sciences, University of York

Karen Whittaker

School of Nursing, University of Central Lancashire

Maria Bryant

Leeds Institute of Clinical Trials Research, University of Leeds

Correspondence concerning this article should be addressed to Nicole Gridley, School of Education, Leeds Beckett University, Leeds, LS6 3QQ
Email: n.gridley@leedsbeckett.ac.uk

Online Resources Table 4.
Summary of the reliability and validity evidence from each study

Measure	Journal Author (Date)	Reliability Evidence	Validity Evidence
AQS	Strayer et al. (1995)	Internal consistency (+/Poor): conducted on each of the eight sub-scales ranging between .19 (Object Use) and .91 (Sociability). Seven of the eight obtained alpha's > .70	Structural validity (+/Fair): Principal Component Analyses (PCA), using seven sub-scales (Object Use was excluded due to poor internal consistency) revealed two factors; 1) Social and, 2) Attachment. The Social factor explained 37.8% variance and was composed of Endurance, Positive Affect, Social Perceptiveness, and Sociability. The second factor, Attachment, accounted for 30.65% variance and was composed using Differential Responsiveness, Proximity/Exploration and the Independence subscales.
	Tarabulsy et al. (1997)	Inter-rater reliability (-/Good): assessed agreement between mother and observer scores (*n*= 79). Results indicated moderate agreement between the raters at two time-points (r = .55) across the eight sub-scales	Convergent/divergent validity (-/Good): correlations with the Parenting Stress Index ranged between r = -.17 (observer security score) and .18 (observer dependency score)
	Teti & McGourty (1996)	Inter-rater reliability (-/Fair): assessed between two trained research staff on seven observations conducted at the same time. Correlations ranged between r = .56 and .93 across the eight sub-scales with the overall security score reaching r = .94.	
CSBS-DP Behaviour Sample	Chambers et al. (2016)	Internal consistency (+/Poor): α's for the US English speaking sample ranged between .87 and .89 at the cluster level, and .86 and .90 at the composite and total score level. For the South African English speaking sample, four of the seven clusters (Sounds, Words,	

Measure	Journal Author (Date)	Reliability Evidence	Validity Evidence
		Understanding and Objects) obtained α's >.70, two (Communication and Gesture) reached ≥.60, whilst the final cluster (Emotion) failed to reach the expected criteria (.22). The overall composite and total scores for this sample ranged between .78 and .90.<br><br>Inter-rater reliability (+/Poor): assessed between coders. Results from a random selection of 25 videotapes - *g* coefficients ranged between .79 and .98 for the cluster scores, .94 to .98 for the composite scores, and .96 for the total score. The overall average consensus reached was .92	
	Eadie et al. (2010)	Internal consistency (+/Excellent): α's only conducted on the three composites and overall total scores. With the exception of the Symbolic composite variable (.44) all other α's exceeded .70<br><br>Inter-rater reliability (+/Good): assessed between a pool of five observers on 10 randomly selected videotaped behaviour samples. Intra-class correlations (ICC's) ranged between .82 and .88 across the three composite and total score values. With the exception of the Speech composite score (.68) all other coefficients were ≥.80	Structural validity (?/Excellent): conducted using Confirmatory Factor Analysis (CFA). A three- (the published speech, social and symbolic composites) -factor model was a better fit to the data with most of the items providing a factor loading of .40 or above. However, the chi square statistic was significant indicating that overall the model could not explain the data. The four-factor model (which separated items relating to the sounds and words clusters into two distinct factors instead of one) was a better fit in comparison to the three-factor model. Despite this, the factor loadings of this model were virtually identical to the three-factor model. These results suggest that the CSBS-DP behaviour sample might be best thought of as a three-factor model comprising the

Measure	Journal Author (Date)	Reliability Evidence	Validity Evidence
			original Social, Speech and Symbolic composite variables.
	Watt et al. (2006)	Internal consistency (+/Poor): α's only conducted on the composite variables which ranged between .86 and .89<br><br>Inter-rater reliability (+/Poor): conducted on a random selection of 20% (n = 32) of the samples scored by each of four raters. g coefficients ranged between .78 and .99 for the items that make up the behaviour sample.	
	Wetherby et al. (2002)	Test-re-test reliability (+/Good): The period between each testing period was four months. Pearson product moment correlation coefficients indicated that all three test-re-test composite, and the overall total scores were highly correlated (range r = .77 to .91). t-tests used to determine the difference between test and retest scores indicated no significant differences for all composite and total score values.<br><br>Inter-rater reliability (+/Good): conducted on five observers coding between 25 and 35 observations at both the cluster, composite and total score level. g coefficients ranged between .76 and .99 at the cluster level, .94 and .99 at the composite level and .96 to .89 at the total score level	

Measure	Journal Author (Date)	Reliability Evidence	Validity Evidence
EAS	Biringen et al. (2005)	Inter-rater reliability (+/Fair): Kappa coefficients between two coders taken on 20% of all cases (*n* = 19) achieved reliability of .92	
	Bornstein et al. (2006a)	Internal consistency (+/Poor): α's were calculated for six scales with one α reported at .91. Individual alpha's are not reported<br><br>Test-re-test reliability: (-/Good) assessed short-term stability over a 1-week period with significant correlations between the two-time points (*p* < .01). The sub-scale, Non-intrusiveness yielded the lowest correlation at .30 and the highest .62. Pairwise *t*-tests indicated no significant difference between scores across the one-week interval. Moreover, Kendall's Tau indicated that approximately 50% of both the mother and infant samples demonstrated stability of their cluster membership.<br><br>Inter-rater reliability (+/Good): ICC's with absolute agreement using a two-way random effects model on 23% (*n* = 8) of the interactions coded by eight observers across all six scales. Four parent (sensitivity, structuring, non-intrusiveness and nonhostility) and two infant (responsiveness and involving) exceeding the acceptable level of .70. The parent scale Non-hostility yielded the lowest agreement amongst coders (.79) whilst Sensitivity) yielded the greatest (.92)	

Measure	Journal Author (Date)	Reliability Evidence	Validity Evidence
	Bornstein et al. (2006b)	Test-re-test reliability (-/Fair): assessed the stability of the EAS between two contexts i.e. the home and the laboratory. Correlations revealed that all six EAS dimensions were highly related across contexts at the $p < .05$ level (range .31 to .64). Non-intrusiveness and Non-hostility were the least related dimensions. Pairwise *t*-tests revealed that home scores were not statistically different from those obtained in the laboratory suggesting stability of the EAS across different contexts.<br><br>Inter-rater reliability (+/Fair): ICC's with absolute agreement using a two-way random effects model for three coders on 20% of the home (n = 11) and 20% (n = 11) of the laboratory cases. Results ranged between .76 and .96. ICC's for Non-intrusiveness and Non-hostility were not computed due to non-normality but percentage agreements were. These ranged between 93 and 100%.	
EC-HOME	Bradley et al. (1994)		Structural validity (+/Fair): using EFA indicated that for the White populations, six factors emerged (Preparation for school, Art/culture influence, Physical environment, Toys and Materials, Verbal Responsiveness and Avoidance of Punishment/Acceptance) explaining 73.1% variance. The authors note that five of these six factors are in accord with the original eight subscales published in the literature. For the African American populations six

Measure	Journal Author (Date)	Reliability Evidence	Validity Evidence
			factors emerged (Preparation for school, Toys, Physical environment, Verbal responsiveness, Avoidance of punishment/acceptance, and Socialisation) explaining 80.4% variance. The authors note that only the socialisation subscale does not marry with those subscales cited in the literature. For the Hispanic ethnic group, eight factors emerged (Toys and materials, Verbal responsiveness, Reading materials, Language stimulation, Preparation for school, Avoidance of punishment/acceptance, Physical environment and Physical affection) explaining 58.9% variance.
	Mundfrom et al. (1993)		Structural validity (+/Fair): for EC-HOME maximum likelihood method of factor analysis extraction with five factors emerging (Toys and materials, Learning stimulation, Physical environment, Responsivity, Lack of punishment/acceptance) explaining 88% variance
	Sugland et al. (1995)	Internal consistency (-/Good): conducted on nine subscales of the EC-HOME (Home learning [now part of Learning Materials and Physical Environment], Acceptance/Punitiveness [now Acceptance], Warmth [now Parental Responsivity], Physical Environment, Modelling, Learning Materials, Language Stimulation, Academic Stimulation and Variety of Stimulation) on three different ethnic populations; African American, Hispanic and European American. Only three of the nine subscales demonstrated α ≥70 using data from the	Convergent/divergent validity (-/Fair): conducted against the Child Behaviour Checklist and the Stanford Binet. Separate regression models were used for the three ethnic groups (White, African American and Hispanic). Results indicated little additional variance explained by scores on EC-HOME over and above the initial demographic variables.

Measure	Journal Author (Date)	Reliability Evidence	Validity Evidence
		overall sample. Individual α's ranged between .36 (Modelling) and .89 (Total score)	
IT-HOME	Bradley et al. (1994)		Structural validity (+/Fair): at the group level (ethnicity) using EFA. Five factors emerged for both White (Responsiveness, Avoidance of punishment/acceptance, toys and materials, involvement and verbal stimulation) and African American ethnic groups (Toys and involvement, avoidance of punishment/acceptance, verbal stimulation, responsiveness and presence of father) explaining 76.4% and 84.9% variance respectively. Seven factors emerged for Hispanic groups (Toys and materials, avoidance of punishment, responsiveness/involvement, verbal stimulation, acceptance, isolation and presence of father) explaining 64.7% of the variance. Overall the IT-HOME appears to be measuring similar constructs in White and African American ethnic groups with some overlap in the Hispanic samples.
	Linver et al. (2004)	Internal consistency (-/Good): α's on seven proposed sub-scales (Parental lack of punitiveness/hostility, Parental support of learning and literacy, Parental warmth, Parental verbal skills, Encouragement of developmental advance, Interior of home, and Exterior of home) where data was available for four different samples. Resulting α's ranged between .39 (Parental lack of hostility) and .77 (Parental warmth) with less than 75% reaching .70	

Measure	Journal Author (Date)	Reliability Evidence	Validity Evidence
	Mitchell & Gray (1981)	Internal consistency (-/Poor): α's for six subscales named; Emotional and verbal responsivity (now Parental responsivity), Avoidance of restriction and punishment (now acceptance of child), Organisation of the environment, Provision of appropriate play materials (now Learning materials), Maternal involvement with child (now parental involvement), and Opportunities for variety in daily stimulation (now variety in experience) at four time points (when the child was four, eight, 12 and 24 months old). Results indicated a range between .00 (Opportunities for variety in daily stimulation at four months old) to .74 (Provision of appropriate play materials at 12 months old) for the six subscales, and .69 to .86 for the total score across the four time points. Only four of the 28 analyses exceeded .70.	
	Mundfrom et al. (1993)		Structural validity (+/Fair): assessed using maximum likelihood method with five common factors emerging (toys and materials, acceptance/lack of punishment, involvement/directly encourage development, responsivity and social savvy/gregariousness) explaining 95.4% of the variance
	Stevens & Bakeman (1985)		Structural validity (?/Fair): PCA of the 45 items of the IT-HOME with three factors emerging; 1) Support for intellectual development (drawing items from provision of appropriate play materials, maternal involvement, opportunities for variety and avoidance of restriction). 2)

Measure	Journal Author (Date)	Reliability Evidence	Validity Evidence
			Verbal responsivity (corresponded to the composite variable emotional and verbal responsivity of the mother). 3) Absence of hostility or annoyance with the child (drew upon items from Avoidance of restriction and punishment and emotional and verbal subscale). The amount of variance explained by this model was not reported
	Tesh & Holditch-Davis (1997)	Internal consistency (+/Fair): found moderate to high levels of internal consistency for four of the six subscales tested (Emotional and verbal responsivity [now Parental responsivity], Avoidance of restriction and punishment [now Acceptance of child], Organisation of the environment, Provision of appropriate play materials [now Learning materials], Maternal involvement [now Parental involvement] and Variety in daily stimulation [now Variety in experience]). Results for these four subscales ranged between α = .61 (Emotional and verbal responsiveness) and .76 (Provision of appropriate play materials). Organisation of the environment and Opportunities for variety demonstrated poor internal consistency (.18 and .40 respectively).	Convergent/divergent validity (?/Good): IT-HOME compared to two other measures; Nursing Child Assessment Teaching Scale (NCATS; Sumner & Spietz, 1994) and naturalistic observations of mother-child behaviour (Holditch-Davies & Thoman, 1988). Correlations with the naturalistic observations ranged between -.69 and .69 with the overall IT-HOME total, whilst correlations with the NCATS were lower, ranging between .24 and .42. The majority of correlations failed to reach .50

NOTE: AQS = Attachment Q-Sort, CSBS-DP = C, EAS = Emotional Availability Scales, EC-HOME = Early Childhood Home Observation Measurement of the Environment, IT-HOME = Infant Home Observation Measurement of the Environment
